# Acoustic Radiation Force Impulse (ARFI) Elastography of Focal Splenic Lesions: Feasibility and Diagnostic Potential

**DOI:** 10.3390/cancers15204964

**Published:** 2023-10-12

**Authors:** Amjad Alhyari, Christian Görg, Suhaib Tahat, Corinna Trenker, Christoph Frank Dietrich, Christina C. Westhoff, Ehsan Safai Zadeh, Hajo Findeisen

**Affiliations:** 1 Department of Gastroenterology, Endocrinology, Metabolism and Infectious Diseases, University Hospital Giessen and Marburg, Philipp University of Marburg, 35033 Marburg, Germany; alhyari@med.uni-marburg.de (A.A.); christian.goerg@uk-gm.de (C.G.); 2 Interdisciplinary Centre of Ultrasound Diagnostics, University Hospital Giessen and Marburg, Philipp University of Marburg, 35033 Marburg, Germany; sohaibtahat@yahoo.com (S.T.); trenker@med.uni-marburg.de (C.T.); ehsan_sz@yahoo.de (E.S.Z.); hajofindeisen@protonmail.com (H.F.); 3 Department of Haematology, Oncology and Immunology, University Hospital Giessen and Marburg, Philipps University of Marburg, 35033 Marburg, Germany; 4 Department Allgemeine Innere Medizin (DAIM), Kliniken Hirslanden Bern, Beau Site, Salem und Permanence, 3013 Bern, Switzerland; 5 Institute of Pathology, University Hospital Giessen and Marburg, Philipps University of Marburg, 35033 Marburg, Germany; westhoff@med.uni-marburg.de; 6 Department of Biomedical Imaging and Imaging-Guided Therapy, Medical University of Vienna, 1090 Vienna, Austria; 7 Department of Internal Medicine, Red Cross Hospital Bremen, 28199 Bremen, Germany

**Keywords:** ARFI elastography, splenic lesions, splenic tumors, ultrasound, spleen stiffness

## Abstract

**Simple Summary:**

Nontraumatic focal splenic lesions (FSL) are rare, and the need for tissue diagnosis must be weighed against the risk of complication after a splenic biopsy. The aim of this retrospective study was to explore the diagnostic potential of acoustic radiation force impulse elastography (ARFI) as a noninvasive method for benign and malignant FSL. Therefore, 34 patients were examined by B-mode ultrasound, contrast-enhanced ultrasound and ARFI. Diagnostic confirmation of FSL was based on histological examination or clinical evaluation with follow-up. Although the lesions’ stiffness was significantly lower than that of the normal splenic parenchyma, regardless of the FSL etiology, the differentiation between benign and malignant FSLs was not possible.

**Abstract:**

Purpose: Nontraumatic focal splenic lesions (FSL) are rare, and the need for tissue diagnosis must be weighed against the very high risk of bleeding after a splenic biopsy. The aim of this study was to explore the feasibility and diagnostic potential of acoustic radiation force impulse (ARFI) elastography as a noninvasive method for different benign and malignant FSLs. No human studies on the elastographic characteristics of FSL exist. Methods: This was a retrospective analysis of 34 patients with FSLs, who underwent abdominal B-mode ultrasound (B-US), contrast-enhanced ultrasound (CEUS), and standardized ARFI examinations between October 2021 and December 2022 at our university hospital. The inclusion criteria were: (i) FSL size ≥ 1 cm; (ii) 10 valid ARFI measurements of the FSL, as well as of the normal splenic parenchyma (NSP) as an in vivo reference; and (iii) diagnostic confirmation of FSL etiology based on histological examination (8/34; 23.5%) or clinical evaluation, which included a clinical and sonographic follow-up (FU), CEUS morphology, and/or morphology on cross-sectional imaging (26/34; 76.5%). CEUS was performed on all patients and the FSLs were classified according to the current guidelines; cross-sectional imaging was available for 29/34 (85.3%). The mean FU duration was 25.8 ± 30.5 months. The mean ARFI velocity (MAV) of the FSL (MAV_L_), the NSP (MAV_P_), and the ratio of the MAV_L_ to the MAV_P_ (MAV_L/P_) were calculated and compared. Results: Of the 34 FSLs, 13 (38.2%) were malignant (mFSL) and 21 (61.8%) were benign (bFSL). The MAV_L_ of all 34 FSLs (2.74 ± 0.71 m/s) was lower than the MAV_P_ (3.20 ± 0.59 m/s), *p* = 0.009, with a mean MAV_L/P_ ratio of 0.90 ± 0.34. No significant differences in the MAV_L_ were observed between the mFSL (2.66 ± 0.67 m/s) and bFSL (2.79 ± 0.75 m/s). There were also no significant differences between the MAV_P_ in patients with mFSL (3.24 ± 0.68 m/s) as compared to that in the patients with bFSL (3.18 ± 0.55 m/s). Likewise, the MAV _L/P_ ratio did not differ between the mFSL (0.90 ± 0.41 m/s) and bFSL (0.90 ± 0.30 m/s) groups. Conclusion: ARFI elastography is feasible in evaluating the stiffness of FSLs. The lesions’ stiffness was lower than that of the NSP, regardless of the FSL etiology. However, differentiation between benign and malignant FSL with the help of this elastographic method does not appear possible. Larger prospective studies are needed to validate these findings.

## 1. Introduction

Focal splenic lesions (FSL) are relatively rare, with an incidence on ultrasound (US) of 0.1–0.2% [1,2]. They can be classified as benign (e.g., infarct, sarcoidosis, hemangioma, hamartoma, lymphangioma, hemangioendothelioma, littoral cell angioma, and focal hematoma) or malignant (e.g., lymphoma, metastases, and angiosarcoma) [3,4]. Most FSLs are discovered incidentally and the majority of them are usually benign [3]. Only 1% of incidentally detected FSLs in patients with no prior oncological history are malignant [5]. Nevertheless, they may pose a diagnostic challenge. One reason for this problem is that such lesions frequently lack a characteristic pattern on different imaging studies, such as B-mode US (B-US) [5,6], contrast-enhanced ultrasound (CEUS) [5,6,7], contrast-enhanced computed tomography (CT) [6,8], magnetic resonance imaging (MRI) [6,9], and positron emission tomography (PET) [6,10]. Secondly, while non-splenic lesions can be further characterized by obtaining tissue biopsies, this is not an easy decision to make when it comes to the spleen, given the well-known higher risk of bleeding [11,12].

Thus, given the histological diversity of FSLs, any unclear lesion requires a further evaluation and/or follow-up (FU), and sometimes an invasive diagnostic approach (a biopsy or even a splenectomy) may be necessary [3].

In 2019, the European Federation for Ultrasound in Medicine and Biology (EFSUMB) published guidelines on the use of elastography in non-hepatic organs [13]. However, due to insufficient scientific evidence, guidance on elastography in splenic lesions is currently unavailable. To the best of our knowledge, there are no human studies which have examined the use of elastography for FSLs. The current study is the first to examine the feasibility and diagnostic potential of acoustic radiation force impulse (ARFI) elastography in human subjects as a quantitative noninvasive tool for characterizing FSLs based on differences in tissue elasticity.

## 2. Patients and Methods

This was a retrospective analysis of 36 consecutive patients with non-traumatic solid FSLs, who underwent abdominal B-US and CEUS examinations of their spleen with an elastographic evaluation using ARFI technology between October 2021 and June 2022 at our tertiary healthcare facility (Marburg university hospital). The study was approved by the local ethics committee (RS 22/13) and conducted in accordance with the amended declaration of Helsinki. Informed consent was obtained from each patient for the ultrasound examination. The inclusion criteria were: 1. Solid FSL ≥ 1 cm in diameter on B-US; 2. Ten valid ARFI measurements of the FSL, as well as 10 measurements of the normal splenic parenchyma (NSP); and 3. Confirmation of the diagnosis by histological examination or an oncologist with over 40 years experience in diagnosing splenic pathologies (C.G.) after at least 3 months follow-up (FU) based on the clinical course, sonographic FU, CEUS morphology, and/or appearance on cross-sectional imaging. FSLs with characteristic B-mode appearances (e.g., simple splenic cysts and wedge-shaped peripheral lesions due to splenic infarction), as well as traumatic splenic lesions, were excluded. In all patients, CEUS morphology was additionally investigated. Cross-sectional imaging (CT, PET-CT, or MRI) was available for 29/34 (85.3%).

### 2.1. B-Mode Ultrasound Examinations

B-mode ultrasounds and CEUS examinations were performed using an Acuson Sequoia 512 GI ultrasound machine (Siemens, Erlangen, Germany) with a 4C1 curved array transducer and a frequency of 4 MHz. With the patient laying supine, the transducer was placed intercostally on the lower part of the left lateral thoracic cage, where the spleen was optimally visualized. Focus and gain were adjusted as needed. The spleen’s long and short axes (in cm) were registered. The FSLs were evaluated for their echogenicity in relation to the nearby splenic tissue as hypoechoic or echogenic, their location in the spleen, and the size of the lesion (largest diameter in cm); in the case of multiple FSLs, the largest lesion was selected for further evaluation with ARFI.

### 2.2. Contrast-Enhanced Ultrasound (CEUS) Examinations

The CEUS examinations were performed with the same transducer in 1.5 MHz contrast-specific mode according to the European Federation of Societies for Ultrasound in Medicine and Biology (EFSUMB) guidelines [14]. The patients received an intravenous bolus of 2.4 mL of second-generation contrast medium (Sono-Vue, Bracco SpA, Milan, Italy), followed by a bolus of 5 mL of NaCl 0.9%. The FSLs were continuously observed over a period of 3–5 min. Videoclips and CEUS images were captured and subsequently analyzed. Contrast enhancement was analyzed during the arterial phase (1–30 s) and the parenchymal phase (>30 s), and interpretation was performed in comparison to adjacent normal splenic tissue as an in vivo reference. All the FSLs were classified according to the guidelines of the World Federation of Societies for Ultrasound in Medicine and Biology (WFSUMB) [5]. All the US and CEUS examinations were performed by a German Society for Ultrasound in Medicine (DEGUM) Level III qualified examiner (C.G., internal medicine) with more than 40 years of ultrasound experience [15].

### 2.3. Acoustic Radiation Force Impulse Examinations

All the elastographic examinations were performed using Siemens Acuson S2000 and Acuson S3000 (Siemens Medical Solutions, Erlangen, Germany) by two qualified investigators (E.S.Z., A.A.) under the supervision and active participation of a DEGUM Level III qualified examiner (C.G., internal medicine). For each patient, two elastographic studies were performed; one of the FSL and the other of the NSP. The ARFI studies were performed as follows: the 6C1 transducer was placed in between the ribs of the left lower part of the thoracic cage, and the depth was adjusted, bringing the area to be measured (FSL or NSP) to the center of the screen. The region of interest (ROI), with dimensions of 10 × 5 mm, was first placed within the FSL. For each measurement, the patient was asked to hold his/her breath in mid-expiration for at least 6 s. The measurement was displayed as velocity (m/s) on the upper corner of the screen. If the lesion moved while being measured, this single reading “shot” was repeated. A total of 10 valid measurements were obtained for each ARFI study [13,16]. An additional ARFI study with 10 measurements was likewise obtained for the NSP. The depths of the measurements and the mean ARFI velocities (MAV) were registered. In total, 2/36 (5.6%) of the patients were excluded due to ARFI measurement failure, so 34 patients were included in the final study analysis.

### 2.4. Statistical Analysis

All the statistical analyses were performed using Excel (Microsoft 365 MSO; Microsoft Corporation, Redmond, WA, USA) and SPSS version 26.0 statistical software (IBM, Armonk, NY, USA). The demographic and biometric data were expressed as mean values ± standard deviations (SD). Statistical evaluation was performed using a Fisher’s exact test for the categorical variables and a Mann–Whitney test for the continuous variables. A *p*-value of <0.05 was defined as significant.

## 3. Results

### 3.1. Demographic Data

Of the 34 study patients, 21 (62%) were males. The mean age was 62 ± 13 years (range 25–86 years). The mean body mass index (BMI) was 26.7 kg/m^2^ (range: 20.3–42.1 kg/m^2^).

### 3.2. Final Diagnosis of FSL

Histological confirmation of an FSL was available for 8/34 (23.5%); for the remaining 26/34 (76.5%) FSLs, the diagnosis was based on the clinical evaluation of an oncologist with over 40 years experience in diagnosing splenic pathologies (C.G.), consisting of at least 3 months of follow-up (FU) based on the clinical course, sonographic FU, CEUS morphology, and/or appearance on cross-sectional imaging, and/or morphological appearance on cross-sectional imaging in accordance with the current diagnostic guidelines [5]. Cross-sectional imaging (CT, PET-CT, or MRI) was available for 29/34 (85.3%). The mean duration of FU was 25.8 ± 30.5 months. Histological specimens were obtained using a US-guided biopsy and examined by two pathologists with experience in splenic and hematological pathologies at a university hospital. Of the 34 FSL, 13 (38.2%) were defined as malignant lesions (mFSL), and 21 (61.8%) were defined as benign (bFSL). Five FSLs were hematological cancers and eight FSLs were non-hematological tumors. An overview of all the disease entities of the FSLs is shown in Table 1.

### 3.3. B-Mode US Data

The mean size of the spleen, as measured by the long and short axes’ diameters (in cm), in the patients with bFSLs (11.36 ± 2.52, and 5.24 ± 1.72, respectively) was similar to that in the patients with mFSLs (12.12 ± 2.07, and 6.10 ± 2.04, respectively), *p* > 0.05. Twenty-five (73.5%) patients had a solitary FSL, and nine (26.5%) patients had multiple FSLs. The anatomical distribution of FSLs within the spleen was as follows; two (5.9%), upper pole; nine (26.5%), lower pole; seven (20.6%), central; seven (20.6%), hilum; one, dome (2.9%); and eight (23.5%), diffuse.

The mean size mFSL was larger than that of bFSL (5.67 ± 4.08 vs. 2.63 ± 1.63 cm), However, this did not reach statistical significance (*p* > 0.06). In total, 13 (38.2%) FSLs were echogenic and 21 (61.8%) were hypoechoic. No significant differences in echogenicity were observed between the mFSLs and bFSLs, *p* > 0.05.

### 3.4. CEUS Data

**Figure 1:**
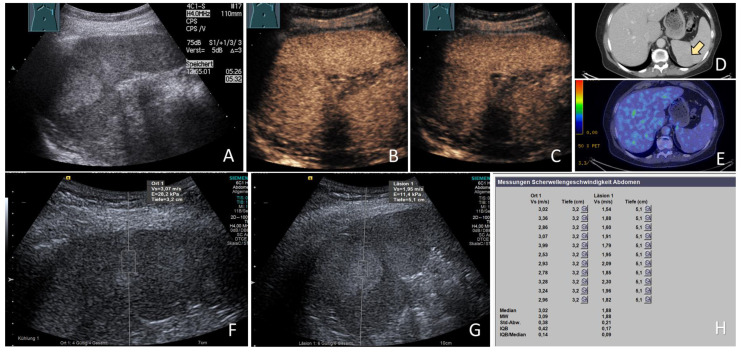
Benign focal splenic lesion (bFSL). 69-year-old asymptomatic female patient with an incidental finding of an FSL; (**A**) B-mode US showing a large echogenic splenic mass, (**B**) on CEUS, the FSL showed an arterial phase isoenhancement with (**C**) mild washout in the late phase after 180 s, (**D**) CT scan of the same FSL (arrow) (courtesy of A. Mahnken, Department of Radiology, University Hospital Marburg), (**E**) PET-CT showing no tracer accumulation within the lesion (courtesy of M. Luster, Department of Nuclear Medicine, University Hospital Marburg), (**F**) US elastography image showing an ARFI measurement of 3.07 m/s within the splenic parenchyma, (**G**) US elastography image showing an ARFI measurement of 1.95 m/s within the lesion, and (**H**) the final ARFI report of the splenic parenchyma (Ort 1) showing a mean ARFI velocity (MW) of 3.09 m/s and of the lesion (Läsion 1) showing a mean ARFI velocity (MW) of 1.88 m/s. A diagnosis of benign vascular lesion (hemangioma) was established and the lesion remained unchanged on follow up at 10 months.

**Figure 2:**
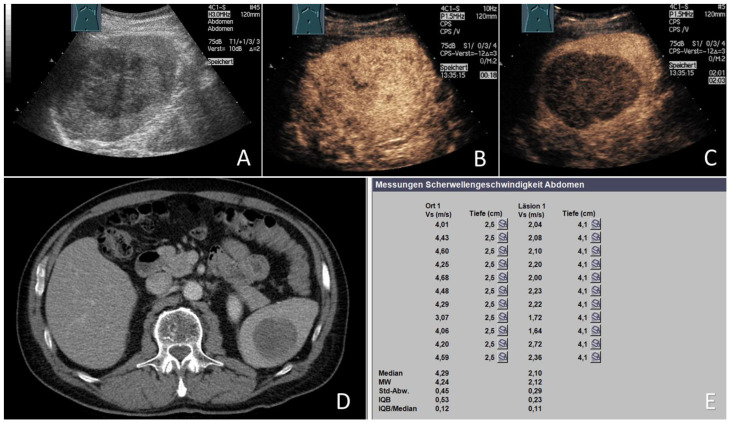
Malignant focal splenic lesion (mFSL). 70-year-old male patient with a known history of metastatic malignant melanoma; (**A**) B-mode US showing a large hypoechoic mass in the center of the spleen, (**B**) on CEUS, the FSL showed an arterial phase isoenhancement with (**C**) marked washout in the late phase, (**D**) CT scan of the same FSL (courtesy of A. Mahnken, Department of Radiology, University Hospital Marburg), and (**E**) the final ARFI report of the splenic parenchyma (Ort 1) showing a mean ARFI velocity (MW) of 4.24 m/s and that of the lesion (Läsion 1) showing a mean ARFI velocity (MW) of 2.12 m/s. The final histology of the US-guided splenic biopsy confirmed the diagnosis of a melanoma metastasis.

The arterial phase demonstrated 8/21 (38.1%) bFSL isoenhancement, 5/21 (23.8%) hyperenhancement, and 8/21 (38.1%) hypoenhancement. In comparison, iso-, hypo-, and hyperenhancement were present in 5/13 (38.5%), 2/13 (15.4%), and 6/13 (46.2%) of mFSLs, respectively. No significant differences were found in the arterial phase enhancement between the bFSLs and mFSLs, *p* > 0.05. On the other hand, during the late phase, 14/21 (66.7%) bFSLs had no or only mild washout (no washout in 6/21 or 28.6% and mild washout in 8/21 or 38.1%), and marked washout was present in 7/21 (33.3%) bFSLs. All 13/13 mFSLs showed marked washout, *p* = 0.001, Figure 1 and Figure 2. Table 2 gives a comprehensive overview of the characteristics of the study cohort and the results of the ultrasound examinations.

### 3.5. ARFI Data

**Figure 3:**
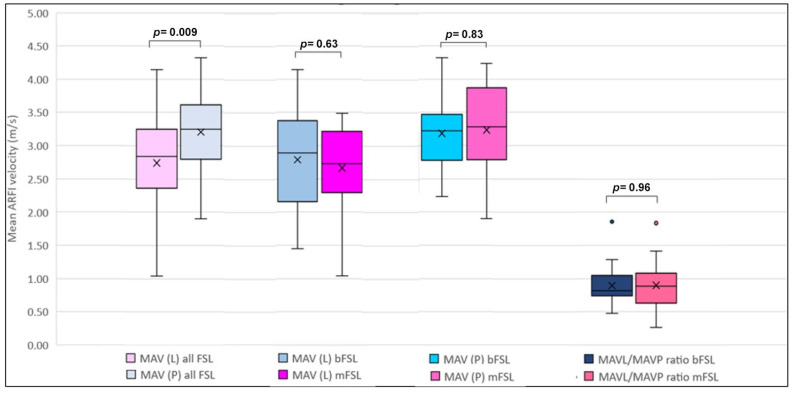
Comparison of mean ARFI velocities of benign and malignant splenic lesions and the background parenchyma of the spleen. The mean is represented by an “X” in each box and the median in different groups is represented by the horizontal line within each box. ARFI: acoustic radiation force impulse; MAV: mean ARFI velocity; MAV (L): MAV of the lesion; MAV (P): MAV of normal appearing splenic parenchyma; bFSL: benign splenic lesion; and mFSL: malignant splenic lesion.

The MAV_L_ of all 34 FSLs (2.74 ± 0.71 m/s) was significantly lower than the MAV_P_ (3.20 ± 0.59 m/s), *p* = 0.009, with a mean MAV_L/P_ ratio of 0.90 ± 0.34. No significant differences in the MAV_L_ were observed between the mFS_L_ (2.66 ± 0.67 m/s) and bFS_L_ (2.79 ± 0.75 m/s)**, Figure 1 and Figure 2**. There were also no significant differences between the MAV_P_ in patients with mFS_L_ (3.24 ± 0.68 m/s), as compared to that in patients with bFS_L_ (3.18 ± 0.55 m/s), *p* > 0.05. Likewise, the MAV_L/P_ ratio did not differ between the mFS_L_ (0.90 ± 0.41 m/s) and the bFS_L_ (0.90 ± 0.30 m/s) groups, *p* > 0.05. A summary of the elastographic results among the benign and malignant FSLs is shown in Figure 3 and Table 3.

## 4. Discussion

US elastography represents a good, non-invasive method for characterizing tissue stiffness with various applications in many organs, including the liver, thyroid, pancreas, kidney, breast, lymph nodes, and prostate [13]. In previous studies, we have shown the feasibility and diagnostic performance of ARFI elastography in differentiating benign from malignant pathologies in the mesentery [17], omentum [18], and lung [19]. There are guidelines regarding the implementation of spleen elastography in patients with liver disease to assess for liver fibrosis, clinically relevant portal hypertension, and the presence of esophageal varices [13]. Additionally, there is some recently reported evidence on the use spleen elastography to differentiate between various etiologies in patients with splenomegaly [20,21]. However, no human studies on the use of elastography to characterize FSLs exist.

There is only one animal study which examined the use of strain elastography in FSLs in dogs [22]. Adler et al. used strain elastography on 22 dogs with 14 FSLs (8 benign, 6 malignant, and 8 normal spleens), and there were no significant differences between the benign and malignant FSLs, nor between the FSLs and NSPs [22].

In the present study, among all 34 patients, the MAV_L_ (2.74 ± 0.71 m/s) was significantly lower than the MAV_P_ (3.20 ± 0.59 m/s), *p* = 0.009. The MAV _L/P_ ratio of all 34 patients was 0.90 ± 0.34. On the other hand, no differences in the MAV_L_ were seen between the mFSLs and bFSLs (2.66 ± 0.67 m/s vs. 2.79 ± 0.75 m/s). There were also no significant differences between the MAV_P_ in patients with mFSLs and those with bFSLs (3.24 ± 0.68 m/s vs. 3.18 ± 0.55 m/s). Likewise, the MAV _L/P_ ratio did not differ significantly between the mFSLs and bFSLs (0.90 ± 0.41 m/s vs. 0.90 ± 0.30 m/s), as shown in Figure 3 and Table 3.

These observations are contrary to neoplastic lesions in other organs, where the stiffness of malignant lesions is often higher than that of benign lesions and the surrounding parenchyma [13,17,18,19]. It is unclear why, in the spleen, as shown in the current study, the stiffness of the FSLs was lower than that of the surrounding NSP. Table 4 shows a comparison of the MAVs in different organs (healthy parenchyma, benign, and malignant solid lesions) as compared to this study.

Due to the rarity of splenic pathologies, there are few data on the histopathological structure of such lesions and their extracellular matrix in comparison to solid neoplastic lesions in other organs [22]. Of note, the degree of normal splenic stiffness in healthy volunteers varies among studies [34,35]. In Table 4, one can notice that the reported stiffness of the spleen is higher than that of the liver and pancreas, but similar to that of the kidney and the thyroid gland (Table 4).

The variable amount of sinusoidal congestion and perisinusoidal fibrosis may be responsible for the increased stiffness of the NSP in some patients [36]. For example, the splenic stiffness in patients with splenomegaly due to portal hypertension was reported to be in the range of 3.27–3.85 m/s [20,21]. It is unclear whether the presence of a bFSL (e.g., vascular tumors or inflammatory lesions with a high blood flow) or profusely vascularized mFSL may contribute to sinusoidal congestion, and hence the increased splenic stiffness of the parenchyma distal to the FSL. On the other hand, whether the phagocytic activity of the spleen in such lesions or the composition of the extracellular tumor matrix are responsible for the lower stiffness of FSLs remains a mere theoretical explanation [37]. Histopathological studies on the geometrical arrangement of the FSL and surrounding splenic parenchyma are needed to explore these theories.

There were some limitations to this study: First: there was no analysis of the interobserver variability, however, various studies have demonstrated the reproducibility of ARFI in different body organs [38]. Second: histological confirmation of FSL was not available for all patients, and their diagnoses were based on CEUS morphology and supported by sonographic FU and/or morphology on cross-sectional imaging, in accordance with current clinical guidelines [22]. Third: despite being used as a measure of reliability of ARFI measurements in other organs, the use of the interquartile range (IQR) and IQR/median ratio as measures of reliability has not been validated in the spleen/splenic lesions. Moreover, obtaining an IQR value of < 60% and IQR/median ratio of < 30% was not possible in all patients. To ensure the validity of the ARFI studies, 10 measurements with a success rate of >60% were obtained for each examination [31]. Fourth: this study is also limited by its retrospective nature and by it being a single-center study from a tertiary university hospital with a relatively small number of patients (keeping in mind, however, the overall rarity of FSLs). Fifth: the feasibility of the ARFI examination also depends on the visualization and detection of FSL in B-mode US, which may be limited due to anatomical conditions, especially in the splenic dome region [39]. Sixth: due to the retrospective nature of this study, the examiners were not blinded to the clinical and radiological data. Therefore, further large prospective studies, preferably comparing with other cross-sectional imaging modalities, are needed to validate our findings.

## 5. Conclusions

In this study, the feasibility of ARFI elastography was demonstrated for evaluating the stiffness of FSLs in relation to NSP. Although the lesions’ stiffness was significantly lower than that of the NSP, regardless of the FSL etiology, the differentiation between benign and malignant FSLs with the help of this elastographic method does not appear possible. These findings add to the mystery of the splenic organ and need further large prospective elastographic and histopathological studies for validation.

## Figures and Tables

**Table 1:** Final diagnoses in 34 study patients. FSL: focal splenic lesion, NEC: neuroendocrine carcinoma.

Group	Benign FSL (n = 21)	Malignant FSL (n = 13)
**Etiology**	Benign vascular tumors (17) Sarcoidosis (2) Chronic infarction (1) Benign fibrous lesion (1)	Malignant lymphoma (5) Solid tumor metastases (8) − Pancreatic NEC (1) − Malignant melanoma (2) − Renal cell carcinoma (1) − Non-small cell lung cancer (3) − Epithelioid hemangioendothelioma (1)

**Table 2:** Characteristics of the study cohort and b-mode ultrasound and CEUS findings.

	Total n (%)	Malignant n (%)	Benign n (%)	*p*-Value
**Focal splenic lesion**	34 (100)	13 (38.2)	21 (61.8)	
**Sex**				*p* = 0.07
**Female**	13 (38.2)	2 (15.4)	11 (84.6)	
**Male**	21 (61.8)	11 (52.4)	10 (47.6)	
**Age**	62.4 ± 12.8	65.8 ± 9.1	60.3 ± 14.2	*p* = 0.3
**Body mass index (in kg/m^2^)**	26.7 ± 4.6	27.6 ± 6.4	26.1 ± 3.1	*p* = 1
**Spleen long axis (in cm)**	11.6 ± 2.6	12.1 ± 2.1	11.4 ± 2.5	*p* = 0.45
**Spleen short axis (in cm)**	5.6 ± 1.9	6.1 ± 2.0	5.2 ± 1.7	*p* = 0.16
**Lesion size (in cm)**	4.0 ± 3.1	2.6 ± 1.6	5.7 ± 4.1	*p* = 0.06
**Number lesions**				*p* = 0.234
**Single**	25 (73.5)	11 (44.0)	14 (56.0)	
**Multiple**	9 (26.5)	2 (22.2)	7 (77.8)	
**B-mode ultrasound**				*p* = 0.015
**Hypoechoic**	21 (61.8)	9 (42.9)	12 (57.1)	
**Isoechoic**	3 (8.8)	3 (100)	0 (0)	
**Hyperechoic**	10 (29.4)	1 (10.0)	9 (90.0)	
**Arterial CEUS enhancement**				*p* = 0.90
**Hypoechoic**	14 (41.2)	6 (42.9)	8 (57.1)	
**Isoechoic**	13 (38.2)	5 (38.5)	8 (61.5)	
**Hyperechoic**	7 (20.6)	2 (28.6)	5 (71.4)	
**CEUS wash out**				*p* = 0.001
**No**	5 (15.2)	0 (0)	5 (100)	
**Mild**	8 (24.2)	0 (0)	8 (100)	
**Marked**	20 (60.6)	13 (65.0)	7 (35.0)	

**Table 3:** Comparison of acoustic radiation force impulse ARFI data obtained from FSLa, defined as benign or malignant lesions, and NSP values among cases of the study population. That of the splenic parenchyma in 34 study patients. FSL: focal splenic lesion, bFSL: benign focal splenic lesion, mFSL: malignant focal splenic lesion, MAV: mean ARFI velocity, NSP: normal splenic parenchyma, and SD: standard deviation.

Group	(n)	Mean ARFI Velocity (m/s) of FSL (MAV_L_)			Average Depth of Measurement for FSL) (Mean ± SD in cm)	Mean ARFI Velocity (m/s) of NSP (MAV_P_)			Average Depth of Measurement for NSP) (Mean ± SD in cm)	MAV _L/P_ Ratio		
		Mean ± SD	Min	Max		Mean ± SD	Min	Max		Mean ± SD	Min	Max
**All FSL**	34	2.74 ± 0.71	1.04	4.14	4.83 ± 1.28	3.20 ± 0.59	1.90	4.32	3.99 ± 1.15	0.90 ± 0.34	0.26	1.86
**bFSL**	21	2.79 ± 0.75	1.45	4.14	3.98 ± 1.15	3.18 ± 0.55	2.23	4.32	3.98 ± 1.12	0.90 ± 0.30	0.47	1.86
**mFSL**	13	2.66 ± 0.57	1.04	3.49	4.78 ± 1.13	3.23 ± 0.68	1.90	4.24	3.99 ± 1.24	0.90 ± 0.41	0.26	1.84

**Table 4:** Comparison of stiffness values obtained using acoustic radiation force impulse (ARFI) elastography in different organs. MAV: mean ARFI velocity, SD: standard deviation.

Organ	MAV± SD in m/s			
	Parenchyma in Healthy Subjects	Parenchyma in Patients with Solid Lesions	Benign Focal Solid Lesions	Malignant Focal Solid Lesions
**Liver**	1.08 ± 0.15 [23,24]	1.35 ± 0.71 [25]	1.51 ± 0.69 [26]	2.31 ± 1.05 [26]
**Thyroid**	2.07 ± 0.44 [27]	2.05 ± 0.43 * [28]	2.01 ± 0.49 [28]	3.94 ± 1.39 [28]
**Pancreas**	1.22 ± 0.36 [29]	1.53 ± 0.45 [30]	3.10 ± 0.40 [31]	3.70 ± 1.00 [31]
**Kidney**	2.21 ± 0.58 [32]	2.28 ± 0.74 [33]	2.19 ± 0.63 [33]	2.99 ± 0.63 [33]
**Spleen**	2.46 ± 0.35 [16]	3.20 ± 0.59) (Present study)	2.79 ± 0.75) (Present study)	2.66 ± 0.57) (Present study)

* Combined mean and SD for benign and malignant nodules. It is also unclear why mFSLs and bFSLs exhibited similar stiffness values.

## Data Availability

The data presented in this study are available in this article.

## References

[1] Caremani M., Occhini U., Caremani A., Tacconi D., Lapini L., Accorsi A., Mazzarelli C. (2013). Focal splenic lesions: US findings. J. Ultrasound.

[2] Warshauer D.M., Hall H.L. (2006). Solitary splenic lesions. Semin. Ultrasound CT MR.

[3] Fasih N., Gulati A., Ryan J., Ramanathan S., Prasad Shanbhogue A.K., McInnes M., Macdonald D.B., Fraser-Hill M.A., Walsh C., Kielar A.Z. (2014). The mysterious organ. Spectrum of focal lesions within the splenic parenchyma: Cross-sectional imaging with emphasis on magnetic resonance imaging. Can. Assoc. Radiol. J..

[4] Ricci Z.J., Oh S.K., Chernyak V., Flusberg M., Rozenblit A.M., Kaul B., Stein M.W., Mazzariol F.S. (2016). Improving diagnosis of atraumatic splenic lesions, part I: Nonneoplastic lesions. Clin. Imaging.

[5] Trenker C., Görg C., Freeman S., Jenssen C., Dong Y., Caraiani C., Ioanițescu E.S., Dietrich C.F. (2021). WFUMB Position Paper-Incidental Findings, How to Manage: Spleen. Ultrasound Med. Biol..

[6] Barat M., Hoeffel C., Aissaoui M., Dohan A., Oudjit A., Dautry R., Paisant A., Malgras B., Cottereau A.S., Soyer P. (2021). Focal splenic lesions: Imaging spectrum of diseases on CT, MRI and PET/CT. Diagn. Interv. Imaging.

[7] Ioanitescu E.S., Copaci I., Mindrut E., Motoi O., Stanciu A.M., Toma L., Iliescu E.L. (2020). Various aspects of Contrast-enhanced Ultrasonography in splenic lesions—A pictorial essay. Med. Ultrason.

[8] Jang S., Kim J.H., Hur B.Y., Ahn S.J., Joo I., Kim M.J., Han J.K. (2018). Role of CT in Differentiating Malignant Focal Splenic Lesions. Korean J. Radiol..

[9] Jang K.M., Kim S.H., Hwang J., Lee S.J., Kang T.W., Lee M.W., Choi D. (2014). Differentiation of malignant from benign focal splenic lesions: Added value of diffusion-weighted MRI. AJR Am. J. Roentgenol..

[10] Metser U., Miller E., Kessler A., Lerman H., Lievshitz G., Oren R., Even-Sapir E. (2005). Solid splenic masses: Evaluation with 18F-FDG PET/CT. J. Nucl. Med..

[11] Safai Zadeh E., Dietrich C.F., Görg C., Bleyl T., Alhyari A., Ignee A., Jenssen C., Trenker C. (2021). Spleen biopsy: “pros and cons” or better “when and when not?”. Z. Gastroenterol..

[12] Strobel D., Bernatik T., Blank W., Will U., Reichel A., Wüstner M., Keim V., Schacherer D., Barreiros A.P., Kunze G. (2015). Incidence of bleeding in 8172 percutaneous ultrasound-guided intraabdominal diagnostic and therapeutic interventions—Results of the prospective multicenter DEGUM interventional ultrasound study (PIUS study). Ultraschall Med..

[13] Săftoiu A., Gilja O.H., Sidhu P.S., Dietrich C.F., Cantisani V., Amy D., Bachmann-Nielsen M., Bob F., Bojunga J., Brock M. (2019). The EFSUMB Guidelines and Recommendations for the Clinical Practice of Elastography in Non-Hepatic Applications: Update 2018. Ultraschall Med..

[14] Sidhu P.S., Cantisani V., Dietrich C.F., Gilja O.H., Saftoiu A., Bartels E., Bertolotto M., Calliada F., Clevert D.A., Cosgrove D. (2018). The EFSUMB Guidelines and Recommendations for the Clinical Practice of Contrast-Enhanced Ultrasound (CEUS) in Non-Hepatic Applications: Update 2017 (Long Version). Ultraschall Med..

[15] Heese F., Görg C. (2006). The value of highest quality ultrasound as a reference for ultrasound diagnosis. Ultraschall Med..

[16] Karlas T., Lindner F., Tröltzsch M., Keim V. (2014). Assessment of spleen stiffness using acoustic radiation force impulse imaging (ARFI): Definition of examination standards and impact of breathing maneuvers. Ultraschall Med..

[17] Alhyari A., Görg C., Dietrich C.F., Kawohl S., Safai Zadeh E. (2022). Diagnostic Performance of Point Shear Wave Elastography (pSWE) Using Acoustic Radiation Force Impulse (ARFI) Technology in Mesenteric Masses: A Feasibility Study. Diagnostics.

[18] Alhyari A., Görg C., Dietrich C.F., Trenker C., Strauch L., Safai Zadeh E. (2022). ARFI elastography of the omentum: Feasibility and diagnostic performance in differentiating benign from malignant omental masses. BMJ Open Gastroenterol..

[19] Alhyari A., Görg C., Dietrich C.F., Trenker C., Ludwig M., Safai Zadeh E. (2022). Diagnostic Performance of Point Shear Wave Elastography Using Acoustic Radiation Force Impulse Technology in Peripheral Pulmonary Consolidations: A Feasibility Study. Ultrasound Med. Biol..

[20] Yalçın K., Demir B. (2021). Spleen stiffness measurement by shear wave elastography using acoustic radiation force impulse in predicting the etiology of splenomegaly. Abdom. Radiol..

[21] Batur A., Alagoz S., Durmaz F., Baran A.I., Ekinci O. (2019). Measurement of Spleen Stiffness by Shear-Wave Elastography for Prediction of Splenomegaly Etiology. Ultrasound Q..

[22] Alder D., Bass D., Spörri M., Kircher P., Ohlerth S. (2013). Does real-time elastography aid in differentiating canine splenic nodules?. Schweiz Arch Tierheilkd.

[23] Kim J.E., Lee J.Y., Kim Y.J., Yoon J.H., Kim S.H., Lee J.M., Han J.K., Choi B.I. (2010). Acoustic radiation force impulse elastography for chronic liver disease: Comparison with ultrasound-based scores of experienced radiologists, Child-Pugh scores and liver function tests. Ultrasound Med. Biol..

[24] Son C.Y., Kim S.U., Han W.K., Choi G.H., Park H., Yang S.C., Choi J.S., Park J.Y., Kim D.Y., Ahn S.H. (2012). Normal liver elasticity values using acoustic radiation force impulse imaging: A prospective study in healthy living liver and kidney donors. J. Gastroenterol. Hepatol..

[25] Heide R., Strobel D., Bernatik T., Goertz R.S. (2010). Characterization of focal liver lesions (FLL) with acoustic radiation force impulse (ARFI) elastometry. Ultraschall Med..

[26] Park H., Park J.Y., Kim D.Y., Ahn S.H., Chon C.Y., Han K.H., Kim S.U. (2013). Characterization of focal liver masses using acoustic radiation force impulse elastography. World J. Gastroenterol..

[27] Sporea I., Vlad M., Bota S., Sirli R.L., Popescu A., Danila M., Sendroiu M., Zosin I. (2011). Thyroid stiffness assessment by acoustic radiation force impulse elastography (ARFI). Ultraschall Med..

[28] Gu J., Du L., Bai M., Chen H., Jia X., Zhao J., Zhang X. (2012). Preliminary study on the diagnostic value of acoustic radiation force impulse technology for differentiating between benign and malignant thyroid nodules. J. Ultrasound Med..

[29] Zaro R., Lupsor-Platon M., Cheviet A., Badea R. (2016). The pursuit of normal reference values of pancreas stiffness by using Acoustic Radiation Force Impulse (ARFI) elastography. Med. Ultrason..

[30] Goertz R.S., Schuderer J., Strobel D., Pfeifer L., Neurath M.F., Wildner D. (2016). Acoustic radiation force impulse shear wave elastography (ARFI) of acute and chronic pancreatitis and pancreatic tumor. Eur. J. Radiol..

[31] Park M.K., Jo J., Kwon H., Cho J.H., Oh J.Y., Noh M.H., Nam K.J. (2014). Usefulness of acoustic radiation force impulse elastography in the differential diagnosis of benign and malignant solid pancreatic lesions. Ultrasonography.

[32] Lee A., Joo D.J., Han W.K., Jeong H.J., Oh M.J., Kim Y.S., Oh Y.T. (2021). Renal tissue elasticity by acoustic radiation force impulse: A prospective study of healthy kidney donors. Medicine.

[33] Göya C., Daggulli M., Hamidi C., Yavuz A., Hattapoglu S., Cetincakmak M.G., Teke M. (2015). The role of quantitative measurement by acoustic radiation force impulse imaging in differentiating benign renal lesions from malignant renal tumours. Radiol. Med..

[34] Ekinci O., Ozgokce M., Turko E., Merter M. (2021). Spleen Stiffness Measurement by Using Shear-Wave Elastography as a Predictor of Progression to Secondary Myelofibrosis. Ultrasound Q..

[35] Hanquinet S., Habre C., Laurent M., Anooshiravani M., Toso S. (2021). Acoustic radiation force impulse imaging: Normal values of spleen stiffness in healthy children. Pediatr. Radiol..

[36] Kondo R., Kage M., Iijima H., Fujimoto J., Nishimura T., Aizawa N., Akiba J., Naito Y., Kusano H., Nakayama M. (2018). Pathological findings that contribute to tissue stiffness in the spleen of liver cirrhosis patients. Hepatol. Res..

[37] Lokmic Z., Lämmermann T., Sixt M., Cardell S., Hallmann R., Sorokin L. (2008). The extracellular matrix of the spleen as a potential organizer of immune cell compartments. Semin. Immunol..

[38] Bob F., Bota S., Sporea I., Sirli R., Petrica L., Schiller A. (2014). Kidney shear wave speed values in subjects with and without renal pathology and inter-operator reproducibility of acoustic radiation force impulse elastography (ARFI)--preliminary results. PLoS ONE.

[39] Stang A., Keles H., Hentschke S., von Seydewitz C.U., Dahlke J., Malzfeldt E., Braumann D. (2009). Differentiation of benign from malignant focal splenic lesions using sulfur hexafluoride-filled microbubble contrast-enhanced pulse-inversion sonography. AJR Am. J. Roentgenol..

